# Health data science course for clinicians: Time to bridge the skills gap?

**DOI:** 10.1177/02676591241291946

**Published:** 2024-10-11

**Authors:** Hafiz Naderi, Yu-Hsuen Yang, Patricia B Munroe, Steffen E Petersen, Mark Westwood, Nay Aung

**Affiliations:** 1 William Harvey Research Institute, NIHR Barts Biomedical Research Centre, 4617 Queen Mary University of London, London, UK; 2 560754 Barts Heart Centre, St Bartholomew’s Hospital, Barts Health NHS Trust, London, UK; 3 Barts Cancer Institute, Centre for Experimental Cancer Medicine, 4617 Queen Mary University of London, London, UK

**Keywords:** big data, data science course, data science, health data science, medical education, R programming

## Abstract

**Background:**

Data science skills are highly relevant for clinicians working in an era of big data in healthcare. However, these skills are not routinely taught, representing a growing unmet educational need. This education report presents a structured short course that was run to teach clinicians data science and the lessons learnt.

**Methods:**

A 1-day introductory course was conducted within a tertiary hospital in London. It consisted of lectures followed by facilitated pair programming exercises in R, an object-oriented programming language. Feedback was collated and participant responses were graded using a Likert scale.

**Results:**

The course was attended by 20 participants. The majority of participants (69%) were in higher speciality cardiology training. While more than half of the participants (56%) received prior training in statistics either through formal taught programmes (e.g., a Master’s degree) or online courses, the participants reported several barriers to expanding their skills in data science due to limited programming skills, lack of dedicated time, training opportunities and awareness. After the short course, there was a significant increase in participants’ self-rated confidence in using R for data analysis (mean response; before the course: 1.69 ± 1.0, after the course: 3.2 ± 0.9, *p* = .0005) and awareness of the capabilities of R (mean response; before the course: 2.1 ± 0.9, after the course: 3.6 ± 0.7, *p* = .0001, on a 5-point Likert scale).

**Conclusion:**

This proof-of-concept study demonstrates that a structured short course can effectively introduce data science skills to clinicians and supports future educational initiatives to integrate data science teaching into medical education.

## Introduction

Data science is a cross-disciplinary field encompassing statistics, mathematics, programming, advanced analytics, and machine learning to extract insights from data. In the era of digitised patient records, high-throughput imaging, multi-omics and large-scale biomedical databases, data science skills have become indispensable for healthcare professionals.^1^ Big data in healthcare holds tremendous potential to improve patient care through applications in predictive modelling, drug and medical device safety surveillance, evaluation of disease heterogeneity, precision medicine and clinical decision support.^2^

The application of data science in cardiovascular disease has already provided demonstrable improvements in patient care. For instance, the use of risk-stratification scores such as GRACE and CHA_2_DS_2_-VASc, based on the large scale analysis of epidemiological databases, are routinely used in practice to identify and manage patients with high risk Non-ST elevation myocardial infarctions or atrial fibrillation, respectively.^3,4^ More recently, in cardiothoracic surgery, a big data approach to existing epidemiological databases such as the American College of Cardiology Foundation (ACCF) National Cardiovascular Data Registry and the Society of Thoracic Surgery (STS) adult cardiac surgery databases has provided practice-changing real-world evidence that patients who have coronary artery bypass grafting have better long term outcomes than those who undergo percutaneous coronary intervention.^5^ The explosion in genomic and molecular data, captured in cardiovascular disease-specific databases such as the American Heart Association’s precision medicine platform and CardioGenBase have enabled genome wide association studies and multi-omic studies of risk factors in cardiovascular pathologies ranging from cardiomyopathy to atherosclerosis.^6,7^ Finally, machine learning techniques promise to significantly improve the sensitivity of cardiac imaging: recent convolutional neural network algorithms such as MOCOnet are able to correct motion artefacts on T1 mapping in cardiovascular magnetic resonance imaging (cMRI). Another deep learning model was able to automatically segment the left ventricular endocardium from the epicardium on cMRI, with implications for precision cardiac surgery.^8^

It is anticipated that 90% of all future NHS jobs will require digital skills to navigate the data-rich healthcare environment.^9^ Despite this, data science skills are currently not incorporated into medical curricula, and there is a growing need to bridge this skills gap for clinicians.^10,11^ This paper presents a structured course to introduce clinicians to fundamental concepts in data science and tools for working effectively with health data.

## Methods

“Health Data Science for Clinicians” was a 1-day, in-person course delivered at St Bartholomew’s Hospital and was accredited by the Royal College of Physicians for Continuing Professional Development. The primary aim of the course was to equip participants with skills required to analyse health data using R, an object-oriented programming language. R was chosen due to its built-in capabilities for statistics and data processing, availability of powerful add-on packages and engaging user community.^12^ This was an introductory course, therefore no prior knowledge in programming was assumed. The course was made available free-of-charge through support from the Barts Guild Charity (https://www.bartsguild.org.uk/).

The course combined lectures and practical hands-on experience of working with data using R and RStudio.^13^ The lectures focused on R syntax, use of Tidyverse packages,^14^ data wrangling techniques and basic statistical principles. These topics were chosen as they form the foundations of clinical data management in R. In particular, the Tidyverse package was felt to provide a more intuitive approach to data wrangling than base R, in keeping with the user-friendly approach to the course. Knowledge gained from the lectures was reinforced by facilitated small group work centred around analysis of the publicly available medical dataset “Heart”.^15^ Pedagogic analysis has demonstrated the effectiveness of including small-group work within large lectures in improving interest in the teaching material.^16^ For the small group work, participants worked in pairs on the same problem using one computer with regular facilitator support. The pair programming approach was chosen as it is an effective pedagogical tool associated with higher student learning and satisfaction.^17^ Finally, the course adopted the spiral approach to learning first described by Harden (1999).^18^ This approach to learning has been recognised for its ability to enable mastery in highly structured bodies of knowledge such as mathematics through the initial use of simple concepts which progressively gain in rigour and abstraction. Concepts in clinical data analysis were introduced in lectures, then recapped in small-group work and finally during the debrief. Within the small-group worksheet, sequential tasks revisited core concepts with increasing levels of difficulty and related new to previous learning. Indeed, the spiral approach has been effectively used to teach health informatics within a Bachelor of Nursing programme in the United Kingdom.^19^ A summary of material covered by the course is outlined in Table 1. Following the course, an online survey was used to obtain feedback using a 5-point Likert grading scale.^20^

**Table 1.** Outline of course material.

Introduction to Data Science in Healthcare	• Introduction to data science as a field of study
	• Applications of data science in healthcare, and reasons to integrate data science into healthcare
Programming in R	• Introduction to R
	• Syntax and basic operations in base R
	• Data classes
	• Control flow and loops
	• Importing and exporting data
	• Packages
Using the Tidyverse package	• “Long” and “short” data
	• Syntax and basic operations in Tidyverse
Statistics with R	• Numeric versus categorical data
	• Predictor and outcome variables
	• Distributions
	• Statistical tests
	• Generating plots
Paired programming exercise	• Facilitated pair work using course worksheet focusing on content covered above
	• Group debrief including suggested solutions and time for questions

## Results

**Figure 1.**
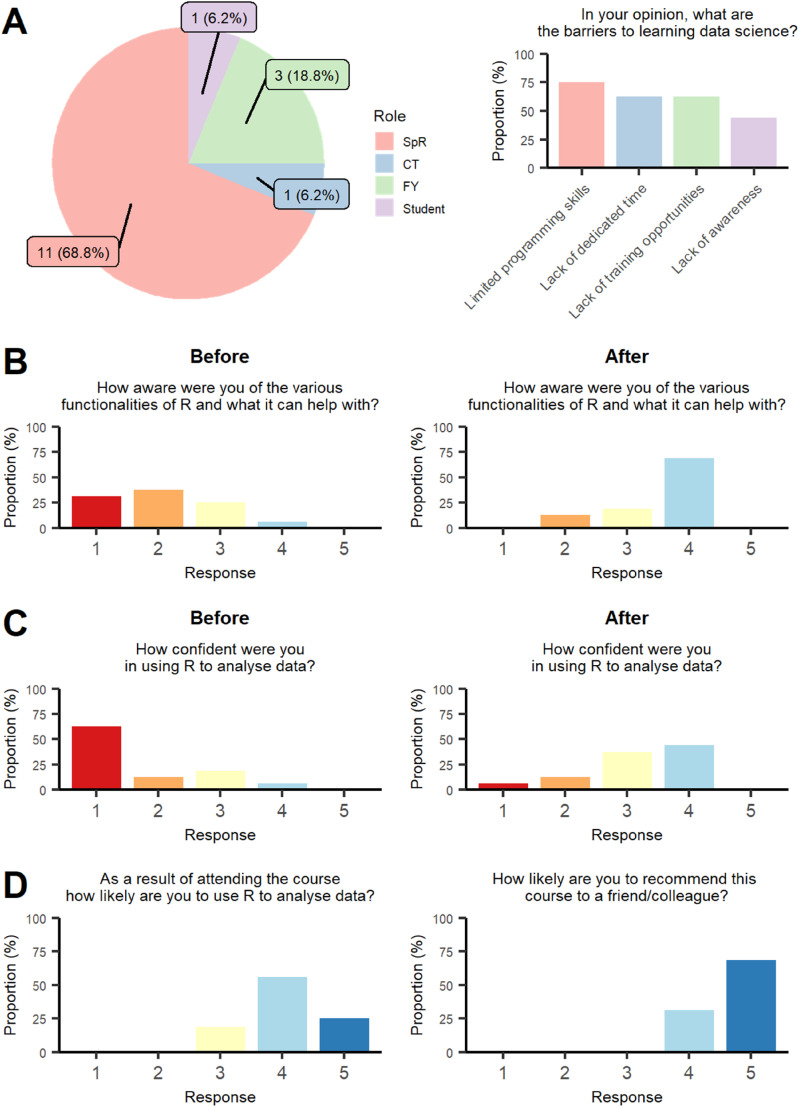
Collated feedback from the Health Data Science for Clinicians course. SpR: Specialist Registrar; CT, Core trainee, FY, Foundation year trainee.

The course proved popular and was fully subscribed within 48 h of registration opening, with 5 participants remaining on the waitlist. 20 participants attended, 16 (80%) of whom provided feedback. Most participants were cardiology trainee registrars (11, 69%) (Figure 1(A)). More than half of the participants had previous training opportunities in data science (9, 56%), ranging from formal teaching such as statistics courses or Master’s degree (3, 19%), to online courses such as Coursera (4, 25%) and informal learning via online resources (2, 13%). Participants cited various barriers to learning data science: limited programming skills at baseline (12, 75%), lack of dedicated time to pursue these skills (10, 63%), few training opportunities (10, 63%) and unawareness of data science in healthcare (7, 44%).

Before attending the course, most participants had limited prior knowledge of the various functionalities of R, self-rating their awareness of these a score of either 1 or 2 (11, 69%). After attendance, most participants rated their awareness of R’s functionalities a score of 4 (11, 69%). Overall, course attendance appeared to significantly increase participants’ self-rated awareness of the capabilities of R for data science (mean response; Before the course: 2.1 ± 0.9, After the course: 3.6 ± 0.7, *p* = .0001) (Figure 1(B)).

In addition, before attending the course, most participants rated their confidence in using R for data analysis lowly, with 12 (75%) participants rating their confidence as either 1 or 2. After the course, the majority of participants rated their confidence as either 3 or 4 (13, 81%). Participating in the course seemed to significantly increase the self-rated confidence of attendees in using R for data analysis (mean response; Before: 1.69 ± 1.0, After: 3.2 ± 0.9, *p* = .0005) (Figure 1(C)).

Overall, participant responses suggested that they found the course highly useful. 15 of 16 (94%) participants stated that they were likely to use R for data analysis in the future as a result of attending the course. All participants would recommend the course to their colleagues (Figure 1(D)).

## Discussion

Data science has become crucial for undertaking research or big data analysis for quality improvement initiatives, and there is an unmet educational need to equip clinicians with these skills. Within the fields of cardiology and cardiothoracic surgery, we anticipate that clinicians will be required to generate and interpret insights from large scale epidemiological and genomic databases, and to understand the algorithms which help to augment cardiac imaging. We present a structured 1-day course aiming to address this issue.

The course was designed to cater to clinicians with no prior experience in data science or programming. As the majority of participants were trainee cardiology registrars within a tertiary centre, the course also aimed to encourage potential future clinical academics to pursue data science in cardiovascular research through a user-friendly aproach. Before the course, more than two-thirds of participants rated their awareness of R’s capabilities and confidence in programming lowly (either 1 or 2 on a Likert scale of 5), suggesting that we had correctly identified our target audience. The content of the course was pitched accordingly to cover fundamentals of data wrangling, and emphasis was placed on the facilitated pair work session to cater to these specific learning needs (Table 1). The responses from our course feedback may be more applicable to early-stage clinical academics who are required to analyse big data, as the course was run within a tertiary centre and mostly attended by participants who were academic clinicians with pre-existing experience in data science.

A major barrier to learning data science raised by participants was their limited programming skills at baseline. During our course, the two main issues encountered by participants were an inability to readily recall the R syntax or to address simple errors through methodical “debugging”. These disincentives which impede further progression are well recognised in teaching computer science, and are particularly problematic for clinicians who may not have undertaken prior quantitative or mathematical degrees.^21,22^ Due to the small group and facilitated nature of our course, we were able to provide immediate input to address these problems and incentivise learning.

Another barrier highlighted was the lack of dedicated time and opportunities, a problem which exists across all stages of training. At present, few medical schools in the United Kingdom currently include data science within their syllabus.^10,23^ For clinicians, the demands of work are often at odds with the focused time, attention and repetition required to learn data science. Without an emphasis on data science within the medical school curricula and post-graduate training, trainees are unlikely to prioritise these skills compared to the vast amount of medical knowledge they are expected to acquire. Integrating data science teaching into medical school curricula through educational modules can help create the conditions conducive to learning data science.^23,24^ Equally, by embedding data science competencies, and providing funding and study leave to attend short courses like ours, healthcare trusts can support trainees acquire data science skills in parallel with their clinical training.

It is notable that within a single day, our course was able to improve participants’ self-rated awareness of R’s functionalities and confidence in health data analysis. These outcomes were achieved in a short timeframe through the effective use of pedagogic theory in the design of the session. There was an intentional focus on foundational concepts in clinical data wrangling, use of small group work with a pair programming approach to bolster didactic lectures, and use of the spiral approach to learning which revisited core data science principles recurrently. This suggests that however compact, data science courses can be highly effective when they are pitched appropriately, provide a clinical context, and apply established pedagogical tools. Short courses are advantageous as they can be integrated into busy clinical rotas.

One of the main limitations of our study is that the effectiveness of our course was assessed by subjective measures rather than improvements on a standardised test. However, as a pilot initiative, it was felt that feedback should be kept simple and open-ended to assess the general efficacy and practicability of the course, and to maximise participant response. Additionally, the nature of the short course and our target audience meant that we were not able to cover more advanced concepts beyond basic data wrangling, such as machine learning and health data privacy. Future work should therefore explore the use of a short courses for clinicians to extend the curricula and assess its efficacy more objectively using standardised tests.

## Conclusions

This proof-of-concept study highlights an unmet educational need in equipping clinicians with data science skills and identifying its barriers. It also demonstrates that a structured short course can be an effective approach to bridge this skills gap. Our initiative supports future work to formally integrate data science education into undergraduate and postgraduate medical training.
